# Supplementary material for: Transfer of modified gut viromes improves symptoms associated with metabolic syndrome in obese male mice
Source: Nat Commun. 2024 Jun 3;15:4704. doi: 10.1038/s41467-024-49152-w (PMC11148109; doi:10.1038/s41467-024-49152-w)
Supplement: Supplementary file 1 — Supplementary Information [file 41467_2024_49152_MOESM1_ESM.pdf]

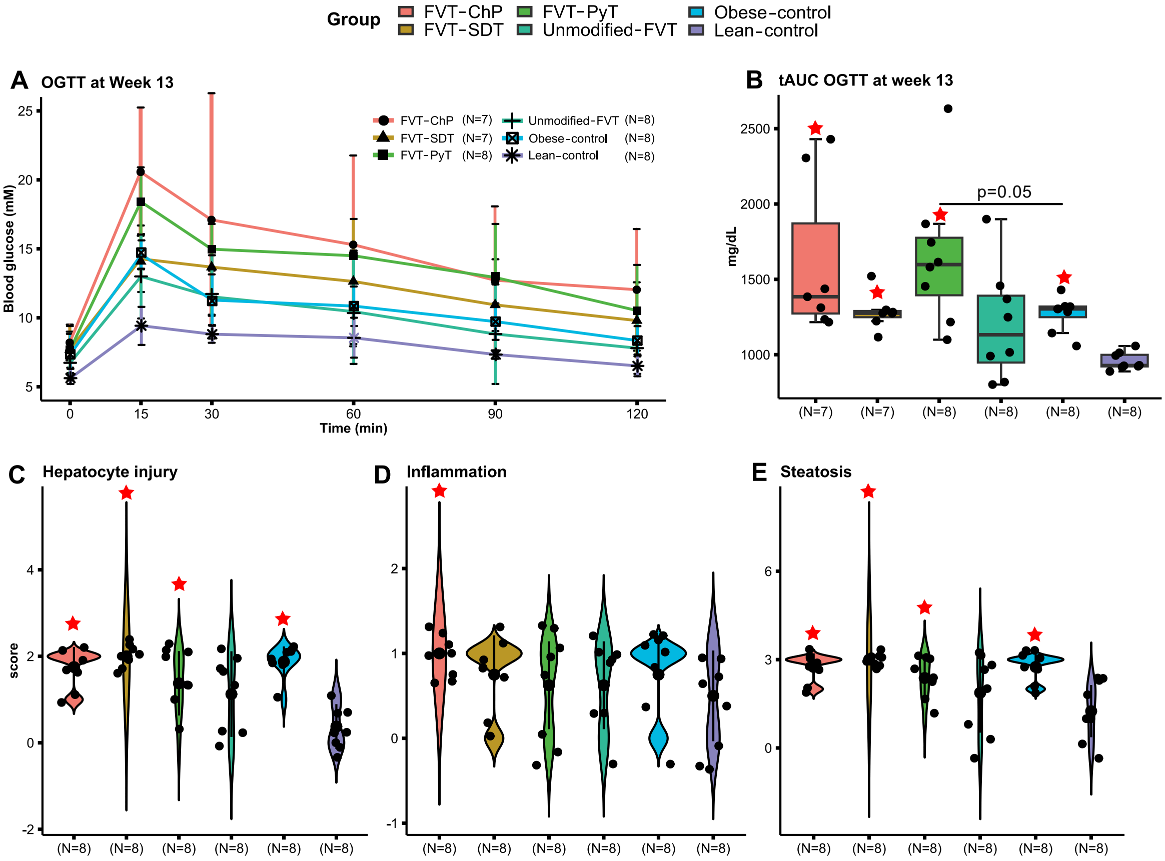

Fig. S1: Overview of mouse phenotypic characteristics after being treated with the different FVTs. A) Oral glucose tolerance tests (OGTT) measured at study week 13 (18 weeks old) (error bars describe mean±SD) B) Total area under the curve (tAUC), and C) to E) sub-categories of MAFLD activity score evaluating hepatocyte injury, inflammation, and steatosis at study week 18 (23 weeks old). The labels of *, **, *** between the FVT treatments and obese control mice represent adjusted p < 0.05, 0.01, 0.001 (two-side Wilcoxon rank-sum test with FDR correction); the unadjusted p is presented in the figure when it is less than 0.05. The red star on top of the boxplot of the treatment represents a p < 0.05 between the treatment and the lean control mice (two-side Wilcoxon rank-sum test). Combined boxplots distributions include median, min, max, 25 and 75 percentiles, and outliers (more than 1.5 IQR).

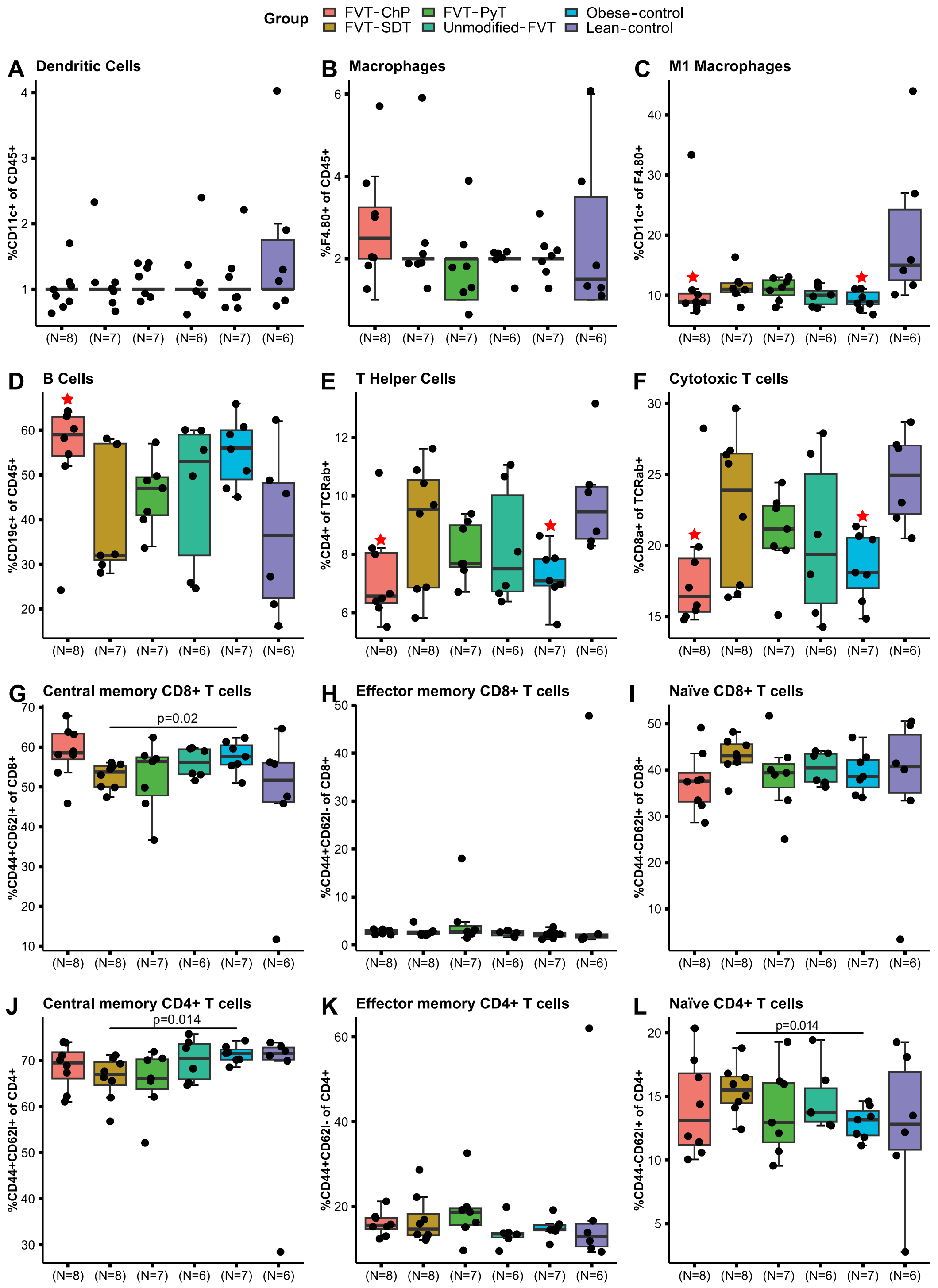

Fig. S2: Proportions of immune cells in the mesenteric lymph node (MLN) at study termination (23 weeks old). A) to L) Show the overall flow cytometric profile in the MLN tissue of the different treatments. The labels of *, **, *** between the FVT treatments and obese control mice represent adjusted $p < 0.05, 0.01, 0.001$ (two-side Wilcoxon rank-sum test with FDR correction), the unadjusted p is presented in figure when it is less than 0.05. The red star on top of the boxplot of the treatment represents a $p < 0.05$ between the treatment and the lean control mice (two-side Wilcoxon rank-sum test). Combined boxplots distributions include median, min, max, 25 and 75 percentiles, and outliers (more than 1.5 IQR).

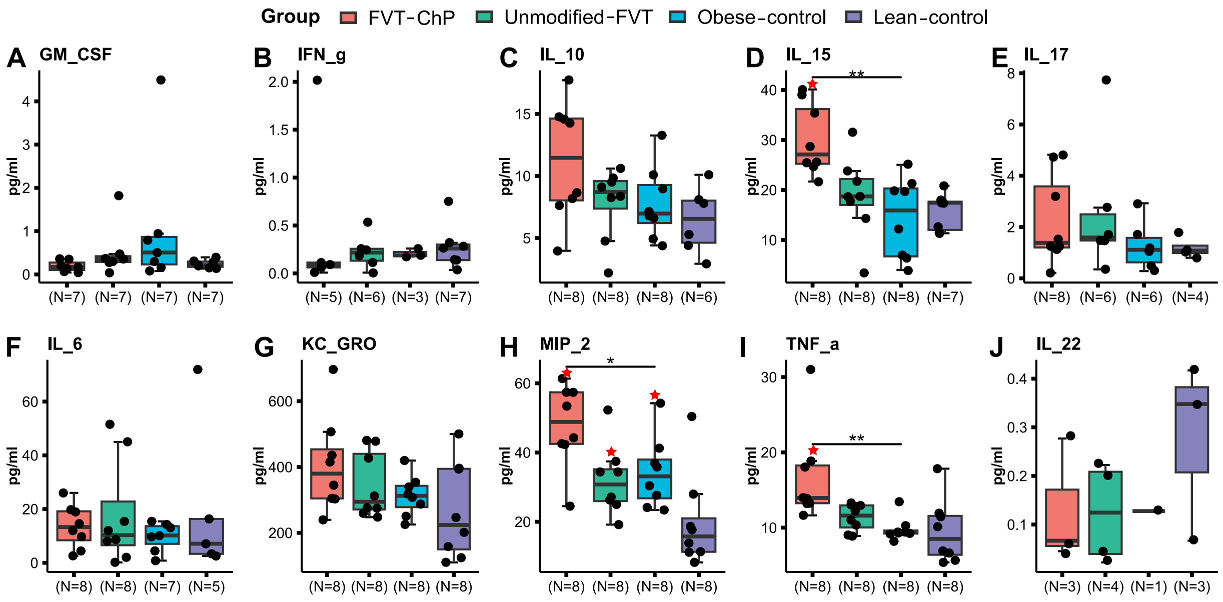

Fig. S3: Cytokine profiles of blood serum sampled at termination (23 weeks old). A) to J) are showing the overall cytokine profile in the mouse blood serum of mice treated with FVT-ChP, unmodified FVT, and the two dietary control groups. The labels of *, **, *** between the FVT treatments and obese control mice represent adjusted p < 0.05, 0.01, 0.001 (two-side Wilcoxon rank-sum test with FDR correction), the unadjusted p is presented in figure when it is less than 0.05. The red star on top of the boxplot of the treatment represents a p < 0.05 between the treatment and the lean control mice (two-side Wilcoxon rank-sum test). Combined boxplots distributions include median, min, max, 25 and 75 percentiles, and outliers (more than 1.5 IQR).

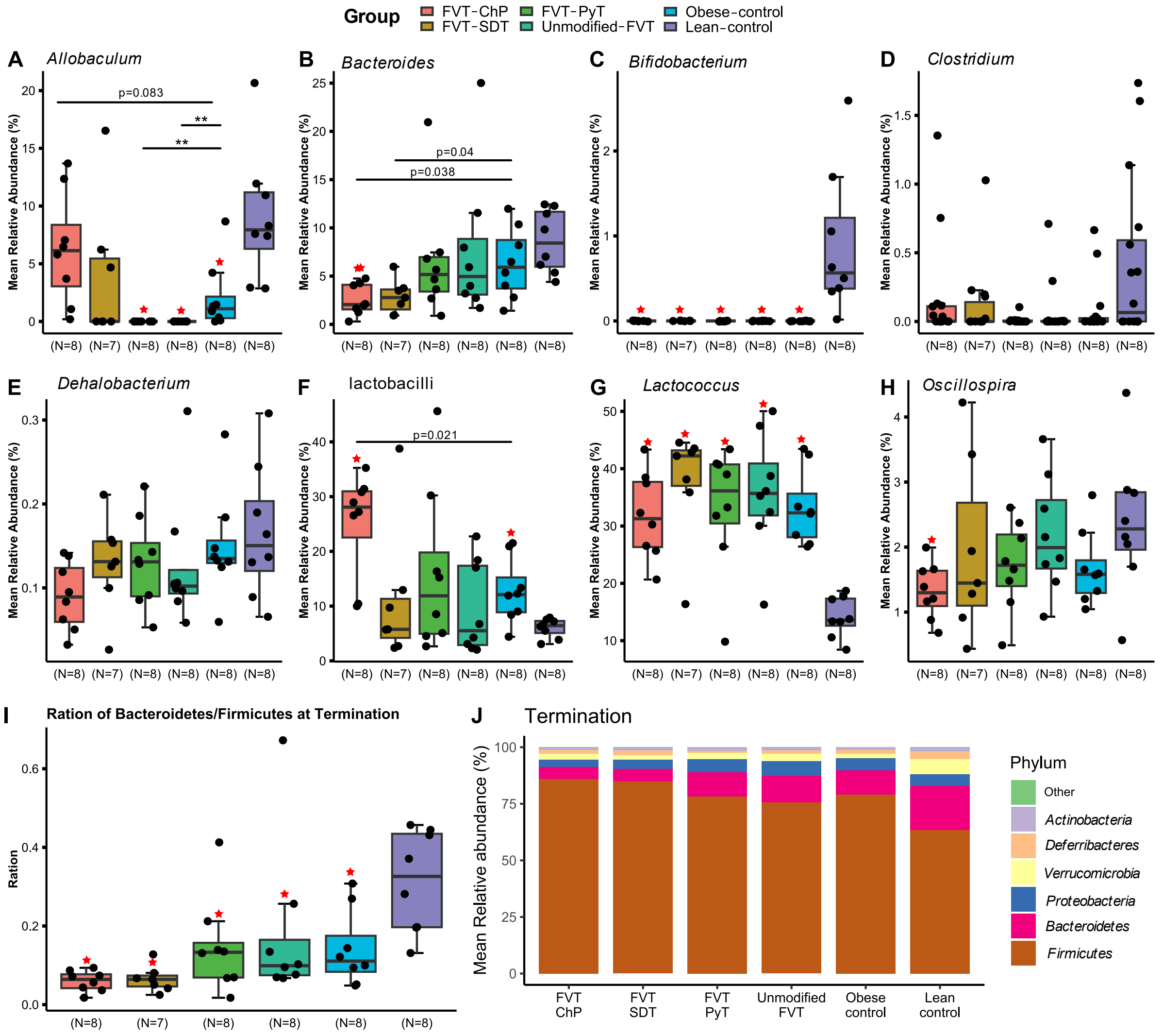

Fig. S4: Relative bacterial abundance at termination (23 weeks old). A) – H) Boxplots showing the relative abundance of selected bacterial taxa. I) Boxplots of the ratio between *Bacteroidetes*:*Firmicutes* that were observed with the different treatment groups. J) Bar plot of phyla distribution based on treatment group. The labels of *, **, *** between the FVT treatments and obese control mice represent adjusted $p < 0.05, 0.01, 0.001$ (two-side Wilcoxon rank-sum test with FDR correction), the unadjusted p is presented in figure when it is less than 0.05. The red star on top of the boxplot of the treatment represents a $p < 0.05$ between the treatment and the lean control mice (two-side Wilcoxon rank-sum test). Combined boxplots distributions include median, min, max, 25 and 75 percentiles, and outliers (more than 1.5 IQR).

See the additional high-resolution PDF Fig. S5

Fig. S5: Heatmap highlighting significant ($p < 0.05$, FDR correction) differences in the differential abundance of viral contigs at termination (23 weeks old).

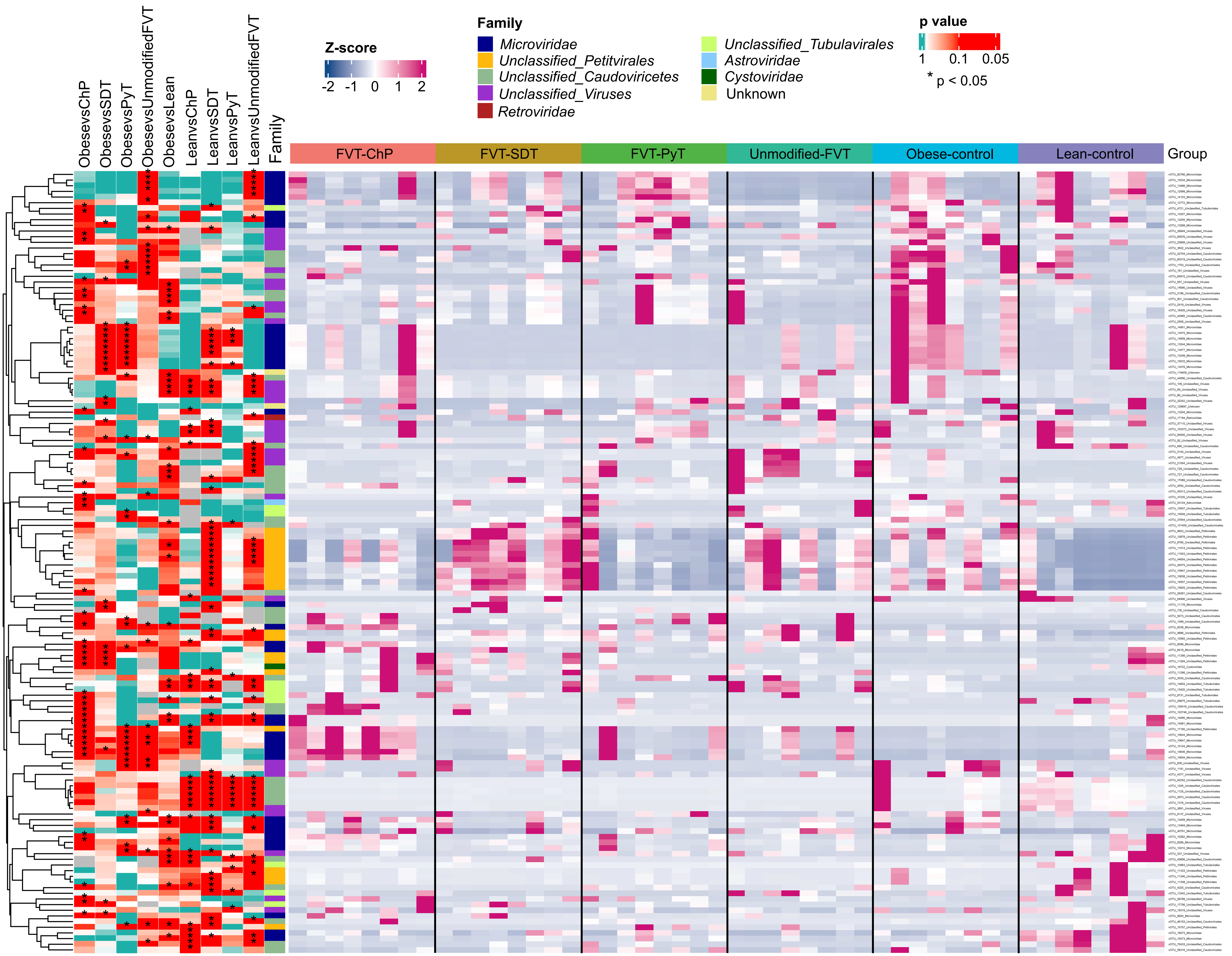

ObesevsChP
ObesevsSDT
ObesevsPyT
ObesevsUnmodifiedFVT
ObesevsLean
LeanvsChP
LeanvsSDT
LeanvsPyT
LeanvsUnmodifiedFVT
Family
Z-score
-2 -1 0 1 2
Family
Microviridae
Unclassified_Petitvirales
Unclassified_Caudoviricetes
Unclassified_Viruses
Retroviridae
Unclassified_Tubulavirales
Astroviridae
Cystoviridae
Unknown
p value
1 0.1 0.05
* p < 0.05
FVT-ChP
FVT-SDT
FVT-PyT
Unmodified-FVT
Obese-control
Lean-control
Group

See the additional high-resolution PDF Fig. S6

Fig. S6: Pairwise Spearman’s correlation between relative abundance of bacterial zOTUs (relative abundance > 0.1%) and viral vOTUs (relative abundance > 0.1%), with the FDR correction for multi-comparison (study week 18, 23 weeks old).

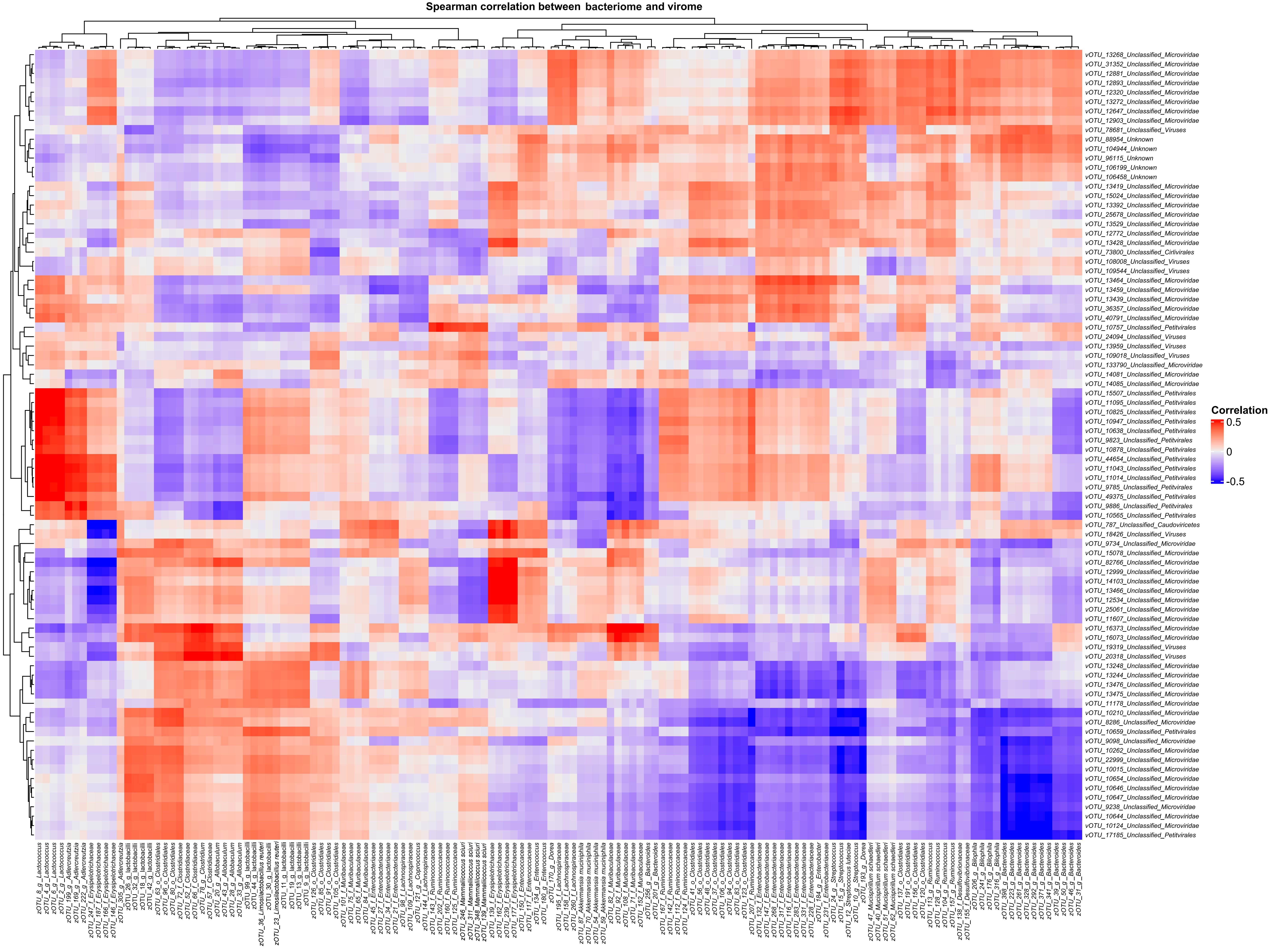
Spearman correlation between bacteriome and virome
Correlation
0.5
0
-0.5
vOTU_13268_Unclassified_Microviridae
vOTU_31352_Unclassified_Microviridae
vOTU_12881_Unclassified_Microviridae
vOTU_12893_Unclassified_Microviridae
vOTU_12320_Unclassified_Microviridae
vOTU_13272_Unclassified_Microviridae
vOTU_12647_Unclassified_Microviridae
vOTU_12903_Unclassified_Microviridae
vOTU_78681_Unclassified_Viruses
vOTU_88954_Unknown
vOTU_104944_Unknown
vOTU_96115_Unknown
vOTU_106199_Unknown
vOTU_106458_Unknown
vOTU_13419_Unclassified_Microviridae
vOTU_15024_Unclassified_Microviridae
vOTU_13392_Unclassified_Microviridae
vOTU_25678_Unclassified_Microviridae
vOTU_13529_Unclassified_Microviridae
vOTU_12772_Unclassified_Microviridae
vOTU_13428_Unclassified_Microviridae
vOTU_73800_Unclassified_Cirlivirales
vOTU_108008_Unclassified_Viruses
vOTU_109544_Unclassified_Viruses
vOTU_13464_Unclassified_Microviridae
vOTU_13459_Unclassified_Microviridae
vOTU_13439_Unclassified_Microviridae
vOTU_36357_Unclassified_Microviridae
vOTU_40791_Unclassified_Microviridae
vOTU_10757_Unclassified_Petitvirales
vOTU_24094_Unclassified_Viruses
vOTU_13959_Unclassified_Viruses
vOTU_109018_Unclassified_Viruses
vOTU_133790_Unclassified_Microviridae
vOTU_14081_Unclassified_Microviridae
vOTU_14085_Unclassified_Microviridae
vOTU_15507_Unclassified_Petitvirales
vOTU_11095_Unclassified_Petitvirales
vOTU_10825_Unclassified_Petitvirales
vOTU_10947_Unclassified_Petitvirales
vOTU_10638_Unclassified_Petitvirales
vOTU_9823_Unclassified_Petitvirales
vOTU_10878_Unclassified_Petitvirales
vOTU_44654_Unclassified_Petitvirales
vOTU_11043_Unclassified_Petitvirales
vOTU_11014_Unclassified_Petitvirales
vOTU_9785_Unclassified_Petitvirales
vOTU_49375_Unclassified_Petitvirales
vOTU_9886_Unclassified_Petitvirales
vOTU_10565_Unclassified_Petitvirales
vOTU_787_Unclassified_Caudoviricetes
vOTU_18426_Unclassified_Viruses
vOTU_9734_Unclassified_Microviridae
vOTU_15078_Unclassified_Microviridae
vOTU_82766_Unclassified_Microviridae
vOTU_12999_Unclassified_Microviridae
vOTU_14103_Unclassified_Microviridae
vOTU_13466_Unclassified_Microviridae
vOTU_12534_Unclassified_Microviridae
vOTU_25061_Unclassified_Microviridae
vOTU_11607_Unclassified_Microviridae
vOTU_16373_Unclassified_Microviridae
vOTU_16073_Unclassified_Microviridae
vOTU_19319_Unclassified_Viruses
vOTU_20318_Unclassified_Viruses
vOTU_13248_Unclassified_Microviridae
vOTU_13244_Unclassified_Microviridae
vOTU_13476_Unclassified_Microviridae
vOTU_13475_Unclassified_Microviridae
vOTU_11178_Unclassified_Microviridae
vOTU_10210_Unclassified_Microviridae
vOTU_8286_Unclassified_Microviridae
vOTU_10659_Unclassified_Petitvirales
vOTU_9098_Unclassified_Microviridae
vOTU_10262_Unclassified_Microviridae
vOTU_22999_Unclassified_Microviridae
vOTU_10015_Unclassified_Microviridae
vOTU_10654_Unclassified_Microviridae
vOTU_10646_Unclassified_Microviridae
vOTU_10647_Unclassified_Microviridae
vOTU_9238_Unclassified_Microviridae
vOTU_10644_Unclassified_Microviridae
vOTU_10124_Unclassified_Microviridae
vOTU_17185_Unclassified_Petitvirales

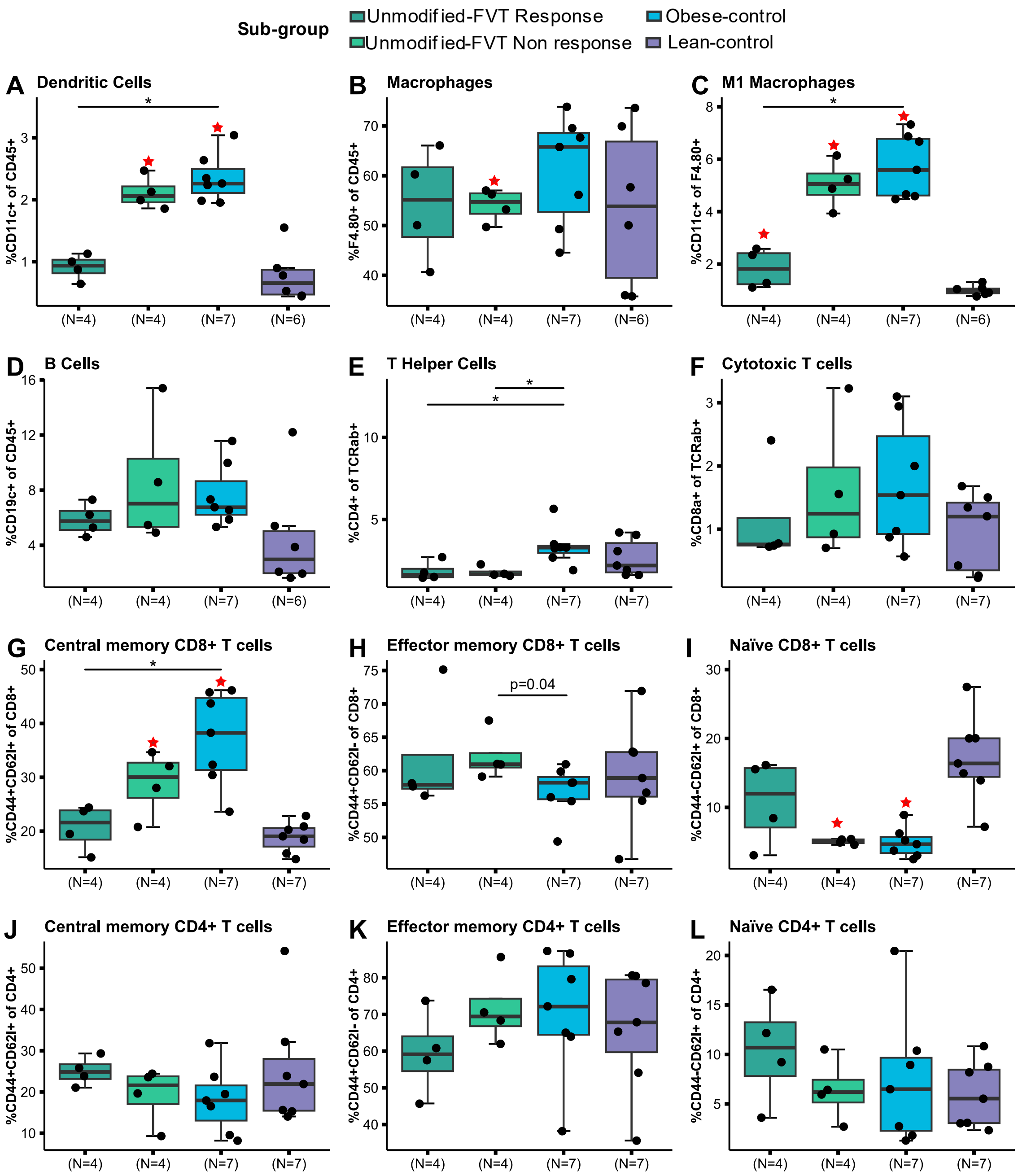

Fig. S7: Proportions of immune cells in adipose tissue of the unmodified FVT treatment stratification analysis at study termination (23 weeks old). A) to L) are showing the overall flow cytometric profile in the mouse adipose tissue. The labels of *, **, *** between the FVT treatments and obese control mice represent adjusted p < 0.05, 0.01, 0.001 (two-side Wilcoxon rank-sum test with FDR correction), the unadjusted p is presented in figure when it is less than 0.05. The red star on top of the boxplot of the treatment represents a p < 0.05 between the treatment and the lean control mice (two-side Wilcoxon rank-sum test). Combined boxplots distributions include median, min, max, 25 and 75 percentiles, and outliers (more than 1.5 IQR).

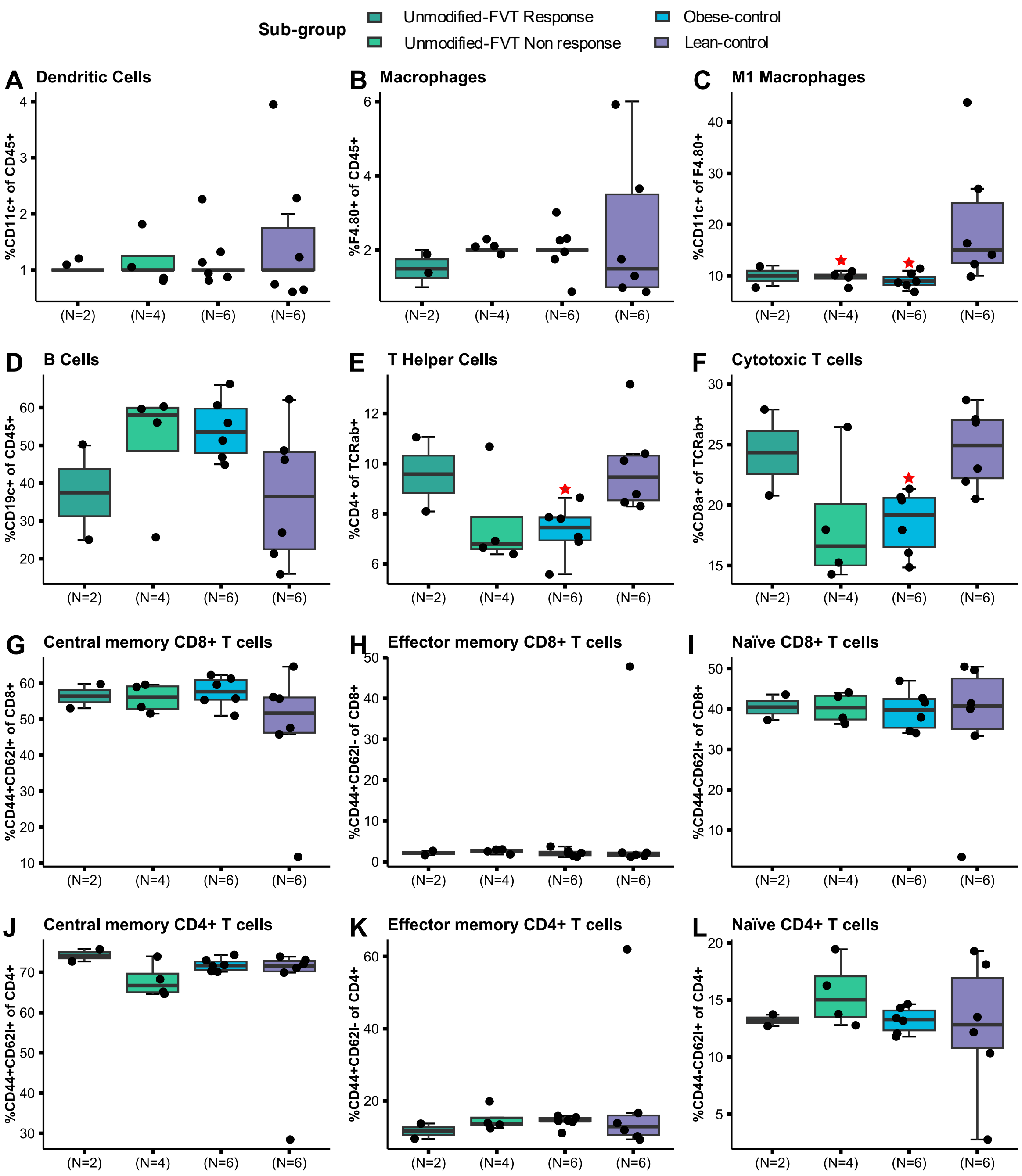

Fig. S8: Proportions of immune cells in mesenteric lymph node (MLN) of the unmodified FVT treatment stratification analysis at study termination (23 weeks old). A) to L) shows the overall flow cytometric profile in the MLN tissue of the different treatments. The labels of *, **, *** between the FVT treatments and obese control mice represent adjusted p < 0.05, 0.01, 0.001 (two-side Wilcoxon rank-sum test with FDR correction), the unadjusted p is presented in figure when it is less than 0.05. The red star on top of the boxplot of the treatment represents a p < 0.05 between the treatment and the lean control mice (two-side Wilcoxon rank-sum test). Combined boxplots distributions include median, min, max, 25 and 75 percentiles, and outliers (more than 1.5 IQR).

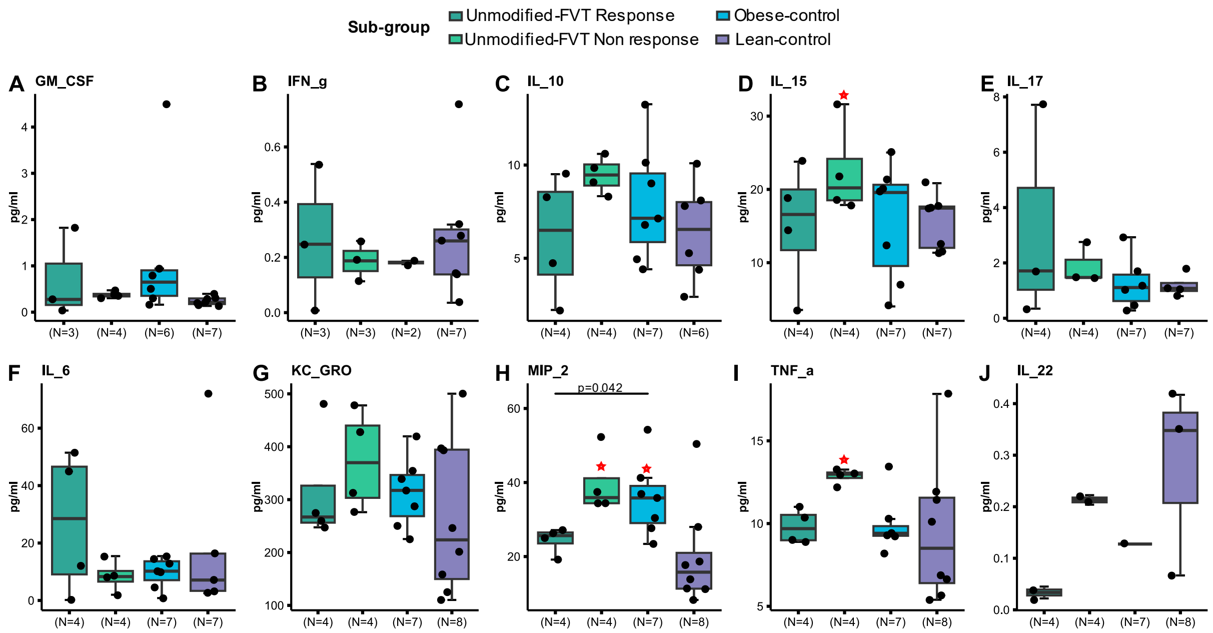

Fig. S9: Cytokine profiles of blood serum of the unmodified FVT treatment stratification analysis at study termination (23 weeks old). A) to J) are showing the overall cytokine profile in the mouse blood serum of mice that responded or not responded on the unmodified FVT, and the two dietary control groups. The labels of *, **, *** between the FVT treatments and obese control mice represent adjusted $p < 0.05, 0.01, 0.001$ (two-side Wilcoxon rank-sum test with FDR correction), the unadjusted p is presented in figure when it is less than 0.05. The red star on top of the boxplot of the treatment represents a $p < 0.05$ between the treatment and the lean control mice (two-side Wilcoxon rank-sum test). Combined boxplots distributions include median, min, max, 25 and 75 percentiles, and outliers (more than 1.5 IQR).

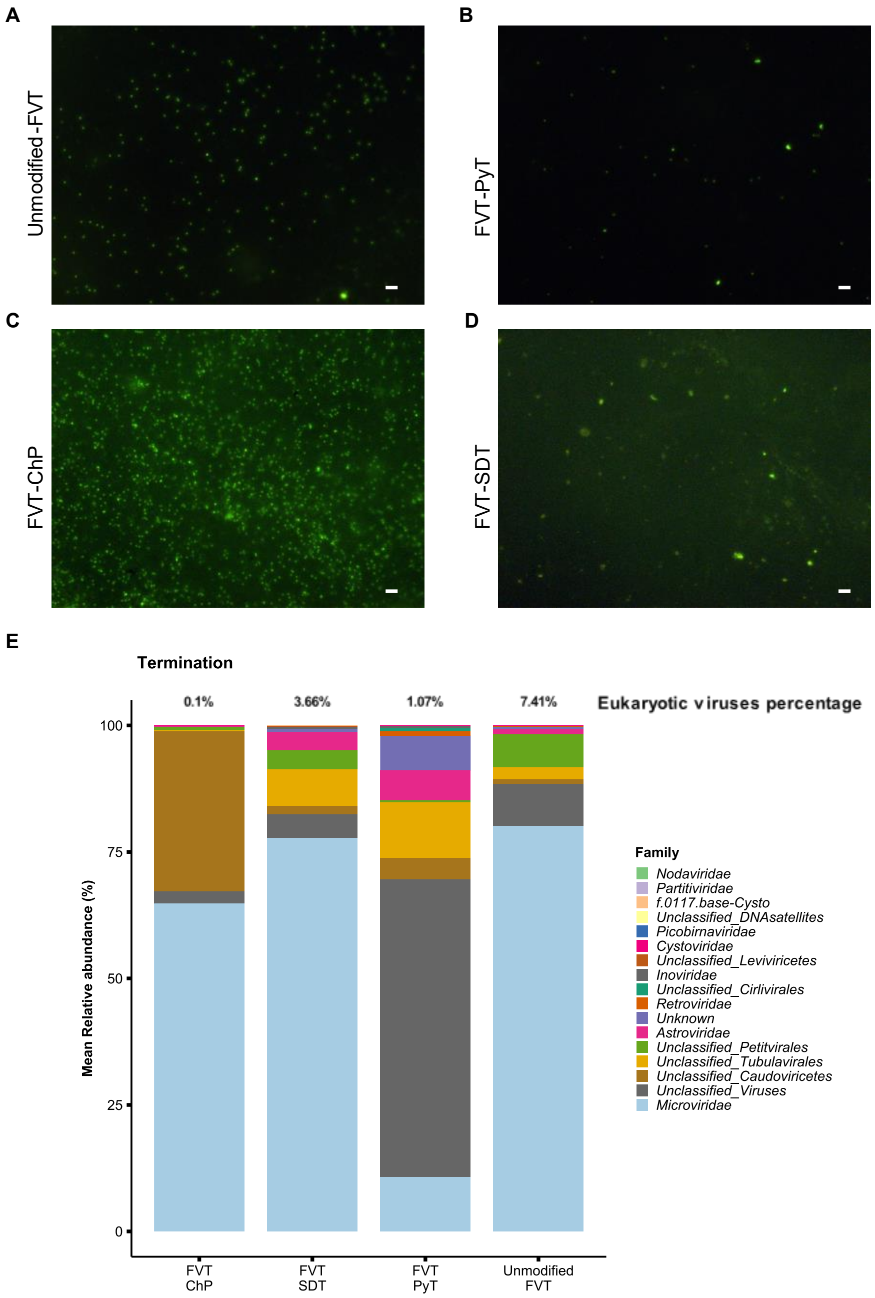

Fig. S10: Overview of the applied FVT viromes. A) – D) Epifluorescence microscopy images of the different applied FVT viromes (before being diluted to similar VLP/mL concentrations) that were stained with SYBR Gold to count VLP/mL (the scale bar is 1 µm). E) Viral taxonomy composition bar plot of the four applied FVTs on the family level including the relative abundance in percentages of eukaryotic viruses in each FVT treatment.

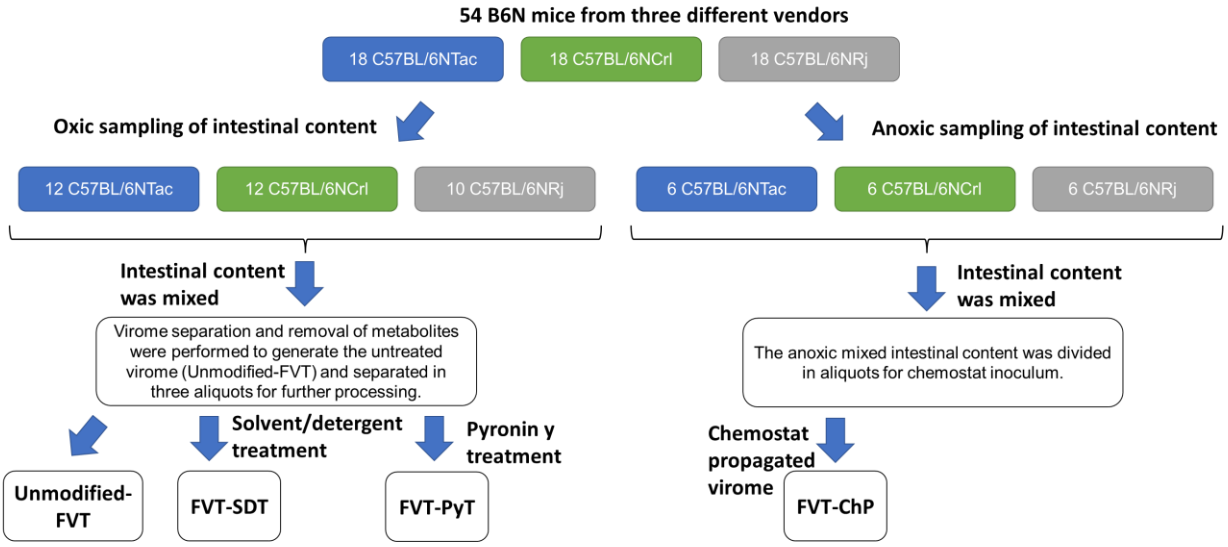

Fig. S11: Flow diagram illustrating the origin of the intestinal donor content and the processing steps to generate the FVTs with different methodologies. Initially, 54 mice from 3 different vendors were sacrificed and their intestinal content from cecum and colon was collected, however, 34 mice were sampled in oxic conditions, while 18 mice were sampled in anoxic conditions to maintain the viability of the strict anaerobic bacterial GM members. Two C57BL/6NRj mice were euthanized due to malocclusion-associated malnutrition. The atmospheric conditions were maintained throughout the process. Regardless of the vendor, the intestinal content was mixed. Virome separation was performed for the oxic-handled fecal mixture and was divided into three aliquots that represented the untreated fecal virome (unmodified FVT), further processing with solvent/detergent treatment (FVT-SDT) or pyronin Y treatment (FVT-PyT). Anoxic handled fecal mixture was used as inoculum for virome propagation in a chemostat setup (FVT-ChP). Also, the FVT-ChP underwent virome separation to remove most metabolites as well as bacteria and other large microbes.

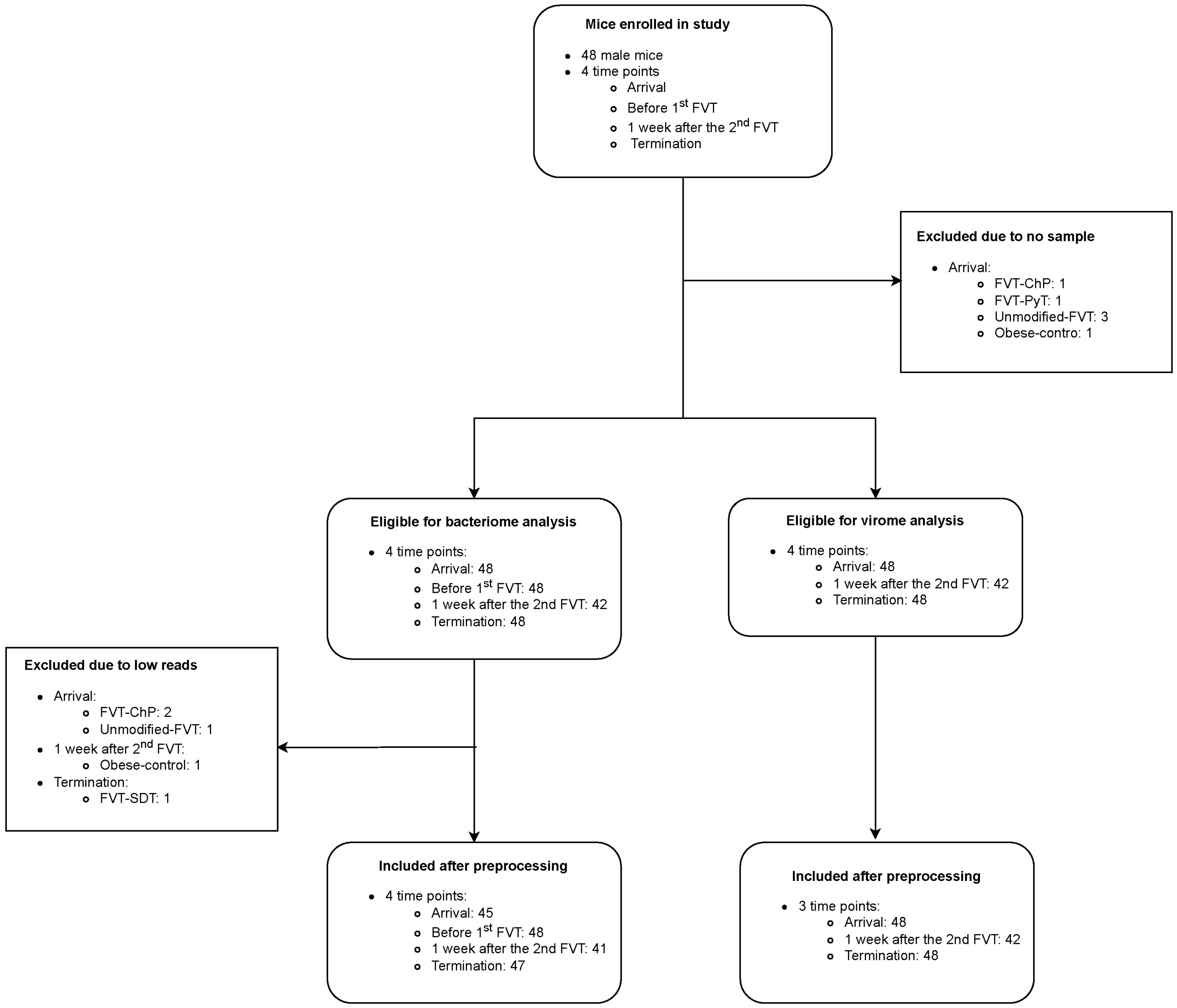

Fig. S12: Flowchart of fecal samples of mice inclusion and exclusion for bacteriome and virome analysis.

Table S1: Overview of the oral glucose tolerance (OGTT) statistics at each time point at study week 13 (18 weeks old). Comparison was done with Wilcoxon rank-sum test (two-side) with FDR correction which was only conducted between the FVT treatments and obese control mice.

	Group1	Group2	n1	n2	p	p.adj
**t0**	FVT-ChP	Obese-control	8	8	0.207	0.528
	FVT-SDT	Obese-control	8	8	0.399	0.528
	FVT-PyT	Obese-control	8	8	0.528	0.528
	Unmodified-FVT	Obese-control	8	8	0.429	0.528
	Obese-control	Lean-control	8	8	0.01	-
	Lean-control	FVT-ChP	8	8	0.006	-
	Lean-control	FVT-SDT	8	8	0.002	-
	Lean-control	FVT-PyT	8	8	0.012	-
	Lean-control	Unmodified-FVT	8	8	0.093	-
**t15**	FVT-ChP	Obese-control	8	8	0.002	0.006
	FVT-SDT	Obese-control	8	8	0.4	0.4
	FVT-PyT	Obese-control	8	8	0.05	0.1
	Unmodified-FVT	Obese-control	8	8	0.279	0.372
	Obese-control	Lean-control	8	8	0.00016	-
	Lean-control	FVT-ChP	8	8	0.012	-
	Lean-control	FVT-SDT	8	8	0.000311	-
	Lean-control	FVT-PyT	8	8	0.003	-
	Lean-control	Unmodified-FVT	8	8	0.015	-
**t30**	FVT-ChP	Obese-control	8	8	0.052	0.185
	FVT-SDT	Obese-control	8	8	0.442	0.589
	FVT-PyT	Obese-control	8	8	0.093	0.185
	Unmodified-FVT	Obese-control	8	8	0.875	0.875
	Obese-control	Lean control	8	8	0.003	-
	Lean-control	FVT-ChP	8	8	0.002	-
	Lean-control	FVT-SDT	8	8	0.00931	-
	Lean-control	FVT-PyT	8	8	0.00931	-
	Lean-control	Unmodified-FVT	8	8	0.002	-
**t60**	FVT-ChP	Obese-control	8	8	0.442	0.673
	FVT-SDT	Obese-control	8	8	0.674	0.674
	FVT-PyT	Obese-control	8	8	0.027	0.108
	Unmodified-FVT	Obese-control	8	8	0.505	0.673
	Obese-control	Lean-control	8	8	0.01	-
	Lean-control	FVT-ChP	8	8	0.045	-
	Lean-control	FVT-SDT	8	8	0.000931	-
	Lean-control	FVT-PyT	8	8	0.003	-
	Lean-control	Unmodified-FVT	8	8	0.002	-
**t90**	FVT-ChP	Obese-control	8	8	0.958	1
	FVT-SDT	Obese-control	8	8	1	1

	FVT-PyT	Obese-control	8	8	0.066	0.264
	Unmodified-FVT	Obese-control	8	8	0.528	1
	Obese-control	Lean-control	8	8	0.013	-
	Lean-control	FVT-ChP	8	8	0.035	-
	Lean-control	FVT-SDT	8	8	0.000155	-
	Lean-control	FVT-PyT	8	8	0.004	-
	Lean-control	Unmodified-FVT	8	8	0.002	-
	FVT-ChP	Obese-control	8	8	0.04	0.16
	FVT-SDT	Obese-control	8	8	0.206	0.275
	FVT-PyT	Obese-control	8	8	0.141	0.275
	Unmodified-FVT	Obese-control	8	8	0.528	0.528
**t120**	Obese-control	Lean-control	8	8	0.006	-
	Lean-control	FVT-ChP	8	8	0.007	-
	Lean-control	FVT-SDT	8	8	0.002	-
	Lean-control	FVT-PyT	8	8	0.016	-
	Lean-control	Unmodified-FVT	8	8	0.003	-

Table S2: Overview of the oral glucose tolerance (OGTT) statistics at each time point at study week 18 (23 weeks old). Comparison was done with Wilcoxon rank-sum test (two-side) with FDR correction which was only conducted between the FVT treatments and obese control mice.

	Group1	Group2	n1	n2	p	p.adj
t0	FVT-ChP	Obese-control	8	8	0.712	0.713
	FVT-SDT	Obese-control	8	8	0.127	0.508
	FVT-PyT	Obese-control	8	8	0.713	0.713
	Unmodified-FVT	Obese-control	8	8	0.293	0.586
	Obese-control	Lean-control	8	8	0.004	-
	Lean-control	FVT-ChP	8	8	0.001	-
	Lean-control	FVT-SDT	8	8	0.013	-
	Lean-control	FVT-PyT	8	8	0.001	-
	Lean-control	Unmodified-FVT	8	8	0.092	-
t15	FVT-ChP	Obese-control	8	8	0.752	1
	FVT-SDT	Obese-control	8	8	0.141	0.564
	FVT-PyT	Obese-control	8	8	1	1
	Unmodified-FVT	Obese-control	8	8	1	1
	Obese-control	Lean-control	8	8	0.005	-
	Lean-control	FVT-ChP	8	8	0.000155	-
	Lean-control	FVT-SDT	8	8	0.000311	-
	Lean-control	FVT-PyT	8	8	0.001	-
	Lean-control	Unmodified-FVT	8	8	0.021	-
t30	FVT-ChP	Obese-control	8	8	0.878	0.878
	FVT-SDT	Obese-control	8	8	0.328	0.878
	FVT-PyT	Obese-control	8	8	0.721	0.878
	Unmodified-FVT	Obese-control	8	8	0.636	0.878
	Obese-control	Lean-control	8	8	0.002	-
	Lean-control	FVT-ChP	8	8	0.00923	-
	Lean-control	FVT-SDT	8	8	0.003	-
	Lean-control	FVT-PyT	8	8	0.003	-
	Lean-control	Unmodified-FVT	8	8	0.103	-
t60	FVT-ChP	Obese-control	8	8	0.021	0.083
	FVT-SDT	Obese-control	8	8	0.462	0.799
	FVT-PyT	Obese-control	8	8	0.875	0.875
	Unmodified-FVT	Obese-control	8	8	0.599	0.799
	Obese-control	Lean-control	8	8	0.000931	-
	Lean-control	FVT-ChP	8	8	0.007	-
	Lean-control	FVT-SDT	8	8	0.007	-
	Lean-control	FVT-PyT	8	8	0.002	-
	Lean-control	Unmodified-FVT	8	8	0.429	-
t90	FVT-ChP	Obese-control	8	8	0.014	0.054
	FVT-SDT	Obese-control	8	8	0.721	0.961

	FVT-PyT	Obese-control	8	8	0.43	0.86
	Unmodified-FVT	Obese-control	8	8	1	1
	Obese-control	Lean-control	8	8	0.000155	-
	Lean-control	FVT-ChP	8	8	0.000915	-
	Lean-control	FVT-SDT	8	8	0.000923	-
	Lean-control	FVT-PyT	8	8	0.001	-
	Lean-control	Unmodified-FVT	8	8	1	-
**t120**	FVT-ChP	Obese-control	8	8	0.171	0.342
	FVT-SDT	Obese-control	8	8	0.269	0.359
	FVT-PyT	Obese-control	8	8	0.114	0.342
	Unmodified-FVT	Obese-control	8	8	0.874	0.874
	Obese-control	Lean-control	8	8	0.002	-
	Lean-control	FVT-ChP	8	8	0.000915	-
	Lean-control	FVT-SDT	8	8	0.000899	-
	Lean-control	FVT-PyT	8	8	0.005	-
	Lean-control	Unmodified-FVT	8	8	0.528	-

Table S3: List showing the included bacterial and phage strains, relevant strain-specific information, and growth conditions.

**Phage**	**DSM ID**	**Family**	**Genome type**	**Bacterial host**	**Strain ID**	**DSM ID**	**Incubation temperature (°C)**	**Media**
C2	In house	*Ceduovirus c2*	dsDNA	*Lactococcus lactis*	MG1363	DSM 4366	30	M17
T4	4505	*Straboviridae*	dsDNA	*Escherichia coli*	Luria	DSM 613	37	LB
PhiX174	4497	*Microviridae*	ssDNA	*Escherichia coli*	PC 0886	DSM 13127	37	BHI
MS2	13767	*Fiersviridae*	ssRNA	*Escherichia coli*	W1485	DSM 5695	37	NZCYM + 2mg/L streptomycin
Phi6	21518	*Cystoviridae*	dsRNA	*Pseudomonas sp.*	HER 1102	DSM 21482	25	TSB

Table S4: Overview of cage-associated effects of phenotypical factors statistics. Comparison was done with Wilcoxon rank-sum test (two-side).

FVT-ChP						
**Phenotypical factors**	**Cage**	**Cage**	**n1**	**n2**	**statistic**	**p**
ewat_mg	6	12	4	4	5	0.486
Weight_gain_perc	6	12	4	4	12	0.343
Cumulative_score	6	12	4	4	8	1
Steatosis	6	12	4	4	8	1
Inflammation	-	-	-	-	-	-
Hepatocyte_injury	6	12	4	4	8	1
OGTT_t0_w18	6	12	4	4	11	0.486
OGTT_t15_w18	6	12	4	4	12	0.343
OGTT_t30_w18	6	12	4	4	12	0.343
OGTT_t60_w18	6	12	4	4	10	0.686
OGTT_t90_w18	6	12	4	4	9.5	0.772
OGTT_t120_w18	6	12	4	4	14	0.114
AUC1_w18	6	12	3	4	8	0.593
OGTT_t0_w13	6	12	4	4	10	0.663
OGTT_t15_w13	6	12	4	4	10	0.686
OGTT_t30_w13	6	12	4	4	10.5	0.561
OGTT_t60_w13	6	12	4	4	9	0.886
OGTT_t90_w13	6	12	4	4	6	0.663
OGTT_t120_w13	6	12	4	4	10.5	0.559
AUC1_w13	6	12	4	3	9	0.4
fat_Dendritic_cells	6	12	4	3	9	0.4
fat_Macrophages	6	12	4	3	11	0.114
fat_M1_macrophages	6	12	4	3	1	0.114
fat_B_cells	6	12	4	3	5	0.857
fat_Cytotoxic_T_cells	6	12	4	3	8	0.593
fat_Th_cells	6	12	4	3	9	0.4
fat_Central_memory_T_cells_CD8	6	12	4	3	3	0.4
fat_Effector_memory_T_cells_CD8	6	12	4	3	4	0.629
fat_Naive_T_cells_CD8	6	12	4	3	8	0.629
fat_Central_memory_T_cells_CD4	6	12	4	3	3	0.4
fat_Effector_memory_T_cells_CD4	6	12	4	3	7	0.857
fat_Naive_T_cells_CD4	6	12	4	3	4	0.629
Bac: Shannon DI - Arrival	12	6	3	3	0	0.1
Bac: Shannon DI – Before 1st FVT	12	6	4	4	8	1
Bac: Shannon DI – 1w after FVT	12	6	4	3	4	0.629
Bac: Shannon DI - Termination	12	6	4	4	5	0.486
Bac: Bray-Curtis - Arrival	12	6	3	3	1.26	0.100
Bac: Bray-Curtis – Before 1st FVT	12	6	4	4	1.53	0.089
Bac: Bray-Curtis – 1w after FVT	12	6	4	3	1.55	0.032
Bac: Bray-Curtis - Termination	12	6	4	4	1.66	0.037
Vir: Shannon DI - Arrival	12	6	4	4	7	0.886
Vir: Shannon DI – 1w after FVT	12	6	4	3	7	0.857
Vir: Shannon DI - Termination	12	6	4	4	11	0.486
Vir: Bray-Curtis - Arrival	12	6	4	4	0.87	0.52
Vir: Bray-Curtis – 1w after FVT	12	6	4	3	1.12	0.36
Vir: Bray-Curtis - Termination	12	6	4	4	1.57	0.139

FVT-SDT						
**Phenotypical factors**	**Cage**	**Cage**	**n1**	**n2**	**statistic**	**p**
ewat_mg	5	11	4	4	0	0.0286
Weight_gain_perc	5	11	4	4	10	0.686
Cumulative_score	5	11	4	4	8	1
Steatosis	-	-	-	-	-	-
Inflammation	-	-	-	-	-	-
Hepatocyte_injury	-	-	-	-	-	-
OGTT_t0_w18	5	11	4	4	6	0.663
OGTT_t15_w18	5	11	4	4	4	0.343
OGTT_t30_w18	5	11	4	4	5	0.486
OGTT_t60_w18	5	11	4	4	2	0.114
OGTT_t90_w18	5	11	4	4	0	0.0286
OGTT_t120_w18	5	11	4	4	2	0.114
AUC1_w18	5	11	4	3	2	0.229
OGTT_t0_w13	5	11	4	4	16	0.0286
OGTT_t15_w13	5	11	4	4	13	0.2
OGTT_t30_w13	5	11	4	4	13	0.2
OGTT_t60_w13	5	11	4	4	9.5	0.772
OGTT_t90_w13	5	11	4	4	15	0.0571
OGTT_t120_w13	5	11	4	4	13	0.183
AUC1_w13	5	11	3	4	8	0.629
fat_Dendritic_cells	5	11	3	4	12	0.0571
fat_Macrophages	5	11	3	4	3	0.4
fat_M1_macrophages	5	11	3	4	8	0.629
fat_B_cells	5	11	3	4	9	0.4
fat_Cytotoxic_T_cells	5	11	3	4	9	0.4
fat_Th_cells	5	11	3	4	8	0.629
fat_Central_memory_T_cells_CD8	5	11	3	4	5	0.857
fat_Effector_memory_T_cells_CD8	5	11	3	4	9	0.4
fat_Naive_T_cells_CD8	5	11	3	4	1	0.114
fat_Central_memory_T_cells_CD4	5	11	3	4	8	0.629
fat_Effector_memory_T_cells_CD4	5	11	3	4	6	1
fat_Naive_T_cells_CD4	5	11	3	4	5	0.857
Bac: Shannon DI - Arrival	11	5	4	4	4	0.343
Bac: Shannon DI – Before 1st FVT	11	5	4	4	6	0.686
Bac: Shannon DI – 1w after FVT	11	5	4	3	2	0.229
Bac: Shannon DI - Termination	11	5	4	3	6	1
Bac: Bray-Curtis - Arrival	11	5	4	4	1.46	0.161
Bac: Bray-Curtis – Before 1st FVT	11	5	4	4	1.82	0.025
Bac: Bray-Curtis – 1w after FVT	11	5	4	3	1.40	0.124
Bac: Bray-Curtis - Termination	11	5	4	3	2.87	0.035
Vir: Shannon DI - Arrival	11	5	4	3	4	0.629
Vir: Shannon DI – 1w after FVT	11	5	3	4	6	1
Vir: Shannon DI - Termination	11	5	3	4	9	0.4
Vir: Bray-Curtis - Arrival	11	5	4	3	1.11	0.278
Vir: Bray-Curtis – 1w after FVT	11	5	3	4	1.78	0.027
Vir: Bray-Curtis - Termination	11	5	3	4	1.45	0.146

FVT-PyT						
**Phenotypical factors**	**Cage**	**Cage**	**n1**	**n2**	**statistic**	**p**
ewat_mg	2	8	4	4	7	0.886
Weight_gain_perc	2	8	4	4	13	0.2
Cumulative_score	2	8	4	4	6.5	0.766
Steatosis	2	8	4	4	7	0.874
Inflammation	2	8	4	4	6	0.608
Hepatocyte_injury	2	8	4	4	7	0.874
OGTT_t0_w18	2	8	4	4	9	0.886
OGTT_t15_w18	2	8	4	4	3	0.2
OGTT_t30_w18	2	8	4	4	4	0.343
OGTT_t60_w18	2	8	4	4	2	0.114
OGTT_t90_w18	2	8	4	4	2.5	0.146
OGTT_t120_w18	2	8	4	4	4	0.309
AUC1_w18	2	8	4	4	2	0.114
OGTT_t0_w13	2	8	4	4	11	0.486
OGTT_t15_w13	2	8	4	4	9	0.886
OGTT_t30_w13	2	8	4	4	7	0.886
OGTT_t60_w13	2	8	4	4	7	0.882
OGTT_t90_w13	2	8	4	4	6	0.686
OGTT_t120_w13	2	8	4	4	5	0.486
AUC1_w13	2	8	4	4	7	0.886
fat_Dendritic_cells	2	8	4	4	8	1
fat_Macrophages	2	8	4	4	11	0.486
fat_M1_macrophages	2	8	4	4	6	0.686
fat_B_cells	2	8	4	4	7	0.886
fat_Cytotoxic_T_cells	2	8	4	4	14	0.114
fat_Th_cells	2	8	4	4	12	0.343

Unmodified-FVT						
**Phenotypical factors**	**Cage**	**Cage**	**n1**	**n2**	**statistic**	**p**
ewat_mg	3	9	4	4	8	1
Weight_gain_perc	3	9	4	4	16	0.0286
Cumulative_score	3	9	4	4	8.5	1
Steatosis	3	9	4	4	10	0.642
Inflammation	3	9	4	4	6	0.608
Hepatocyte_injury	3	9	4	4	9	0.874
OGTT_t0_w18	3	9	4	4	15	0.0571
OGTT_t15_w18	3	9	4	4	12	0.343
OGTT_t30_w18	3	9	4	4	13	0.2
OGTT_t60_w18	3	9	4	4	11	0.486
OGTT_t90_w18	3	9	4	4	16	0.0294
OGTT_t120_w18	3	9	4	4	16	0.0286
AUC1_w18	3	9	3	4	9	0.4
OGTT_t0_w13	3	9	4	4	10	0.663
OGTT_t15_w13	3	9	4	4	8	1
OGTT_t30_w13	3	9	4	4	9	0.886
OGTT_t60_w13	3	9	4	4	7	0.886
OGTT_t90_w13	3	9	4	4	8	1
OGTT_t120_w13	3	9	4	4	9	0.886
AUC1_w13	3	9	4	4	8	1
fat_Dendritic_cells	3	9	4	4	10	0.686
fat_Macrophages	3	9	4	4	13	0.2
fat_M1_macrophages	3	9	4	4	9	0.886
fat_B_cells	3	9	4	4	9	0.886
fat_Cytotoxic_T_cells	3	9	4	4	12	0.343
fat_Th_cells	3	9	4	4	12	0.343

fat_Central_memory_T_cells_CD8	2	8	4	4	7	0.886	fat_Central_memory_T_cells_CD8	3	9	4	4	14	0.114
fat_Effector_memory_T_cells_CD8	2	8	4	4	14	0.114	fat_Effector_memory_T_cells_CD8	3	9	4	4	12	0.343
fat_Naive_T_cells_CD8	2	8	4	4	2	0.114	fat_Naive_T_cells_CD8	3	9	4	4	4	0.343
fat_Central_memory_T_cells_CD4	2	8	4	4	5	0.486	fat_Central_memory_T_cells_CD4	3	9	4	4	11	0.486
fat_Effector_memory_T_cells_CD4	2	8	4	4	13	0.2	fat_Effector_memory_T_cells_CD4	3	9	4	4	7	0.886
fat_Naive_T_cells_CD4	2	8	4	4	2	0.114	fat_Naive_T_cells_CD4	3	9	4	4	9	0.886
Bac: Shannon DI - Arrival	2	8	4	4	15	0.0571	Bac: Shannon DI - Arrival	3	9	4	3	4	0.629
Bac: Shannon DI – Before 1st FVT	2	8	4	4	13	0.2	Bac: Shannon DI – Before 1st FVT	3	9	4	4	7	0.886
Bac: Shannon DI – 1w after FVT	2	8	4	3	3	0.4	Bac: Shannon DI – 1w after FVT	3	9	3	2	1	0.4
Bac: Shannon DI - Termination	2	8	4	4	2	0.114	Bac: Shannon DI - Termination	3	9	4	4	4	0.343
Bac: Bray-Curtis - Arrival	2	8	4	4	1.82	0.027	Bac: Bray-Curtis - Arrival	3	9	4	3	3.13	0.036
Bac: Bray-Curtis – Before 1st FVT	2	8	4	4	2.09	0.02	Bac: Bray-Curtis – Before 1st FVT	3	9	4	4	1.93	0.024
Bac: Bray-Curtis – 1w after FVT	2	8	4	3	1.43	0.067	Bac: Bray-Curtis – 1w after FVT	3	9	3	2	1.37	0.1
Bac: Bray-Curtis - Termination	2	8	4	4	2.34	0.027	Bac: Bray-Curtis - Termination	3	9	4	4	2.00	0.049
Vir: Shannon DI - Arrival	2	8	4	4	10	0.686	Vir: Shannon DI - Arrival	3	9	4	4	11	0.486
Vir: Shannon DI – 1w after FVT	2	8	4	3	4	0.629	Vir: Shannon DI – 1w after FVT	3	9	3	2	3	1
Vir: Shannon DI - Termination	2	8	4	4	9	0.886	Vir: Shannon DI - Termination	3	9	4	4	9	0.886
Vir: Bray-Curtis - Arrival	2	8	4	4	2.79	0.022	Vir: Bray-Curtis - Arrival	3	9	4	4	2.62	0.028
Vir: Bray-Curtis – 1w after FVT	2	8	4	3	1.52	0.147	Vir: Bray-Curtis – 1w after FVT	3	9	3	2	3.13	0.1
Vir: Bray-Curtis - Termination	2	8	4	4	1.28	0.246	Vir: Bray-Curtis - Termination	3	9	4	4	1.55	0.179
**Obese-control**							**Lean-control**						
**Phenotypical factors**	**Cage**	**Cage**	**n1**	**n2**	**statistic**	**p**	**Phenotypical factors**	**Cage**	**Cage**	**n1**	**n2**	**statistic**	**p**
ewat_mg	4	10	4	4	3	0.2	ewat_mg	1	7	4	4	2	0.114
Weight_gain_perc	4	10	4	4	16	0.0286	Weight_gain_perc	1	7	4	4	10	0.686
Cumulative_score	4	10	4	4	9	0.874	Cumulative_score	1	7	4	4	6	0.655
Steatosis	4	10	4	4	12	0.181	Steatosis	1	7	4	4	3	0.161
Inflammation	4	10	4	4	4	0.181	Inflammation	1	7	4	4	8	1
Hepatocyte_injury	4	10	4	4	10	0.453	Hepatocyte_injury	1	7	4	4	10	0.608
OGTT_t0_w18	4	10	4	4	5	0.465	OGTT_t0_w18	1	7	4	4	8	1
OGTT_t15_w18	4	10	4	4	6.5	0.772	OGTT_t15_w18	1	7	4	4	12	0.343
OGTT_t30_w18	4	10	4	4	10	0.686	OGTT_t30_w18	1	7	4	4	14.5	0.0814
OGTT_t60_w18	4	10	4	4	13	0.2	OGTT_t60_w18	1	7	4	4	6	0.663
OGTT_t90_w18	4	10	4	4	13	0.2	OGTT_t90_w18	1	7	4	4	4	0.343
OGTT_t120_w18	4	10	4	4	14	0.104	OGTT_t120_w18	1	7	4	4	10	0.663
AUC1_w18	4	10	4	4	12	0.343	AUC1_w18	1	7	4	4	9	0.886
OGTT_t0_w13	4	10	4	4	13.5	0.146	OGTT_t0_w13	1	7	4	4	6.5	0.772
OGTT_t15_w13	4	10	4	4	5	0.486	OGTT_t15_w13	1	7	4	4	10	0.686
OGTT_t30_w13	4	10	4	4	6	0.686	OGTT_t30_w13	1	7	4	4	6.5	0.772
OGTT_t60_w13	4	10	4	4	5	0.486	OGTT_t60_w13	1	7	4	4	13.5	0.146
OGTT_t90_w13	4	10	4	4	8.5	1	OGTT_t90_w13	1	7	4	4	11	0.465
OGTT_t120_w13	4	10	4	4	9	0.885	OGTT_t120_w13	1	7	4	4	0	0.0294
AUC1_w13	4	10	4	4	6	0.686	AUC1_w13	1	7	4	4	13	0.2
fat_Dendritic_cells	4	10	4	4	6	0.686	fat_Dendritic_cells	1	7	2	4	0	0.133
fat_Macrophages	4	10	4	4	16	0.0286	fat_Macrophages	1	7	2	4	8	0.133
fat_M1_macrophages	4	10	4	4	2	0.114	fat_M1_macrophages	1	7	2	4	4	1
fat_B_cells	4	10	4	4	4	0.343	fat_B_cells	1	7	2	4	5	0.8
fat_Cytotoxic_T_cells	4	10	4	4	8	1	fat_Cytotoxic_T_cells	1	7	3	4	4	0.629
fat_Th_cells	4	10	4	4	16	0.0286	fat_Th_cells	1	7	3	4	8	0.629
fat_Central_memory_T_cells_CD8	4	10	4	4	16	0.0286	fat_Central_memory_T_cells_CD8	1	7	3	4	9	0.4
fat_Effector_memory_T_cells_CD8	4	10	4	4	8	1	fat_Effector_memory_T_cells_CD8	1	7	3	4	0	0.0571
fat_Naive_T_cells_CD8	4	10	4	4	1	0.0571	fat_Naive_T_cells_CD8	1	7	3	4	12	0.0571
fat_Central_memory_T_cells_CD4	4	10	4	4	5	0.486	fat_Central_memory_T_cells_CD4	1	7	3	4	4	0.629
fat_Effector_memory_T_cells_CD4	4	10	4	4	11	0.486	fat_Effector_memory_T_cells_CD4	1	7	3	4	9	0.4
fat_Naive_T_cells_CD4	4	10	4	4	5	0.486	fat_Naive_T_cells_CD4	1	7	3	4	1	0.114
Bac: Shannon DI - Arrival	10	4	4	4	7	0.886	Bac: Shannon DI - Arrival	1	7	4	4	7	0.886
Bac: Shannon DI – Before 1st FVT	10	4	4	4	12	0.343	Bac: Shannon DI – Before 1st FVT	1	7	4	4	11	0.486
Bac: Shannon DI – 1w after FVT	10	4	3	4	5	0.857	Bac: Shannon DI – 1w after FVT	1	7	4	4	7	0.886
Bac: Shannon DI - Termination	10	4	4	4	6	0.686	Bac: Shannon DI - Termination	1	7	4	4	1	0.0571
sBac: Bray-Curtis - Arrival	10	4	4	4	2.00	0.028	Bac: Bray-Curtis - Arrival	1	7	4	4	2.35	0.026
Bac: Bray-Curtis – Before 1st FVT	10	4	4	4	2.35	0.026	Bac: Bray-Curtis – Before 1st FVT	1	7	4	4	2.61	0.031
Bac: Bray-Curtis – 1w after FVT	10	4	3	4	1.59	0.035	Bac: Bray-Curtis – 1w after FVT	1	7	4	4	1.98	0.022
Bac: Bray-Curtis - Termination	10	4	4	4	2.27	0.034	Bac: Bray-Curtis - Termination	1	7	4	4	3.57	0.024
Vir: Shannon DI - Arrival	10	4	4	4	8	1	Vir: Shannon DI - Arrival	1	7	4	3	2	0.229
Vir: Shannon DI – 1w after FVT	10	4	3	4	6	1	Vir: Shannon DI – 1w after FVT	1	7	3	4	6	1
Vir: Shannon DI - Termination	10	4	4	4	8	1	Vir: Shannon DI - Termination	1	7	3	4	5	0.857
Vir: Bray-Curtis - Arrival	10	4	4	4	1.78	0.033	Vir: Bray-Curtis - Arrival	1	7	4	3	3.21	0.004
Vir: Bray-Curtis – 1w after FVT	10	4	3	4	2.07	0.025	Vir: Bray-Curtis – 1w after FVT	1	7	3	4	0.91	0.531
Vir: Bray-Curtis - Termination	10	4	4	4	2.47	0.033	Vir: Bray-Curtis - Termination	1	7	3	4	2.45	0.064
